# When human guanylate-binding proteins meet viral infections

**DOI:** 10.1186/s12929-021-00716-8

**Published:** 2021-03-05

**Authors:** Rongzhao Zhang, Zhixin Li, Yan-Dong Tang, Chenhe Su, Chunfu Zheng

**Affiliations:** 1 Department of Immunology, School of Basic Medical Sciences, Fujian Medical University, Fuzhou, Fujian China; 2 State Key Laboratory of Veterinary Biotechnology, Harbin Veterinary Research Institute of Chinese Academy of Agricultural Sciences, Harbin, Heilongjiang China; 3 Fuzhou Medical College of Nanchang University, Fuzhou, Jiangxi China; 4 Department of Microbiology, Immunology and Infectious Diseases, University of Calgary, Calgary, AB Canada

**Keywords:** GBPs, Antiviral roles, Innate immunity, IFN-I, Virus

## Abstract

Innate immunity is the first line of host defense against viral infection. After invading into the cells, pathogen-associated-molecular-patterns derived from viruses are recognized by pattern recognition receptors to activate the downstream signaling pathways to induce the production of type I interferons (IFN-I) and inflammatory cytokines, which play critical functions in the host antiviral innate immune responses. Guanylate-binding proteins (GBPs) are IFN-inducible antiviral effectors belonging to the guanosine triphosphatases family. In addition to exerting direct antiviral functions against certain viruses, a few GBPs also exhibit regulatory roles on the host antiviral innate immunity. However, our understanding of the underlying molecular mechanisms of GBPs' roles in viral infection and host antiviral innate immune signaling is still very limited. Therefore, here we present an updated overview of the functions of GBPs during viral infection and in antiviral innate immunity, and highlight discrepancies in reported findings and current challenges for future studies, which will advance our understanding of the functions of GBPs and provide a scientific and theoretical basis for the regulation of antiviral innate immunity.

## Introduction

Viral infection triggers the recognition of pathogen-associated molecular patterns or danger-associated molecular patterns by host pattern recognition receptors (PRRs), including Toll-like receptors, retinoic-acid-inducible gene I-like receptors, and also certain DNA sensors. These PRRs then initiate rapid activation of downstream signaling cascades leading to the production of type I interferons (IFN-I) [1–4] and eventually interferon-stimulated genes (ISGs), which exert direct antiviral and immune‐regulatory functions [5]. Intriguingly, some ISGs are directly induced by viral infection independent of IFN-I production [6–8].

Among the most abundant ISGs, IFN-inducible guanosine triphosphatases (GTPases) are a conserved superfamily, including myxoma resistance proteins, immunity-related GTPases, Guanylate-binding proteins (GBPs), and very large inducible GTPases [9]. Recent studies have uncovered that GBPs show vital roles in host defense against diverse pathogens, including bacteria, protozoa, and viruses. However, the underlying molecular mechanisms of GBPs in the host antiviral innate immunity have not been fully understood. Here we present an updated overview of current findings on GBPs' roles during viral infections, hoping to understand further the relationship between GBPs and the host antiviral innate immunity.

## The structure and functions of GBPs

GBPs are anciently conserved and widely distributed in eukaryotes. To date, seven human GBPs have been identified [10, 11]. GBPs share a common structure with a globular N-terminal large GTPase (LG) domain followed by a helical domain, further subdivided into a middle domain and a C-terminal α12/13-domain [12, 13]. The LG domain is involved in GTPase and GDPase activity and retains the main biochemical functions of GBPs. Once binding to the nucleotide, GBPs will undergo oligomerization and subsequently mediate the hydrolysis of guanosine-5′-triphosphate (GTP) to guanosine-5′-diphosphate (GDP) and guanosine-5′-monophosphate (GMP) [14], while the hydrolysis of GTP mediates structural rearrangement of GBPs, which is important for the proper localization and the formation of multimers [14–19], and the multimers may be deposited on pathogen-associated membranes to constitute sensory platforms, which induces antimicrobial immunity [19–22]. Whereas the helical domain allows for the interactions of protein–lipid as well as protein–protein; for example, GBP1 binds p62-Ub for delivery to light chain 3B (LC3B)^+^ membranes and engulfment, GBP1 participates along with GBP7 in the trafficking of mono-ubiquitinated protein cargo into autolysosomes, and GBP7 brings ATG4B for LC3b^+^ membrane elongation and closure around the cargo [23].

Some GBPs, such as GBP1, GBP2, and GBP5, have C-terminal CaaX motifs, which can be isoprenylated to provide anchorage to endomembranes organelles [18, 24, 25]. GBPs are typically expressed in various cells and tissues under physiological conditions, except for GBP6 and GBP7, which are constitutively expressed mainly in the oropharynx and liver, respectively [11]. As for the subcellular distribution, GBPs are predominately localized in the cytoplasm, with GBP1 displaying a diffuse or a granular pattern, or both distribution in the cytoplasm; GBP3 localizing in the cytoplasm diffusely; GBP5 being concentrated in the perinuclear region and co-localized with the Golgi apparatus. However, GBP2 and GBP4 are distributed throughout both the nucleus and the cytoplasm. Stimulation with IFN-γ and aluminum fluoride-induced a Golgi translocation of GBP1 and GBP2, but not GBP3, GBP4, or GBP5, and the trafficking may facilitate the association of GBPs with membranes and the formation of protein multimers [26, 27].

## Antiviral GBPs

Accumulating evidence has demonstrated that GBPs play specialized roles in host defenses against intracellular pathogens, including numerous viruses.

### GBP1

**Fig. 1:**
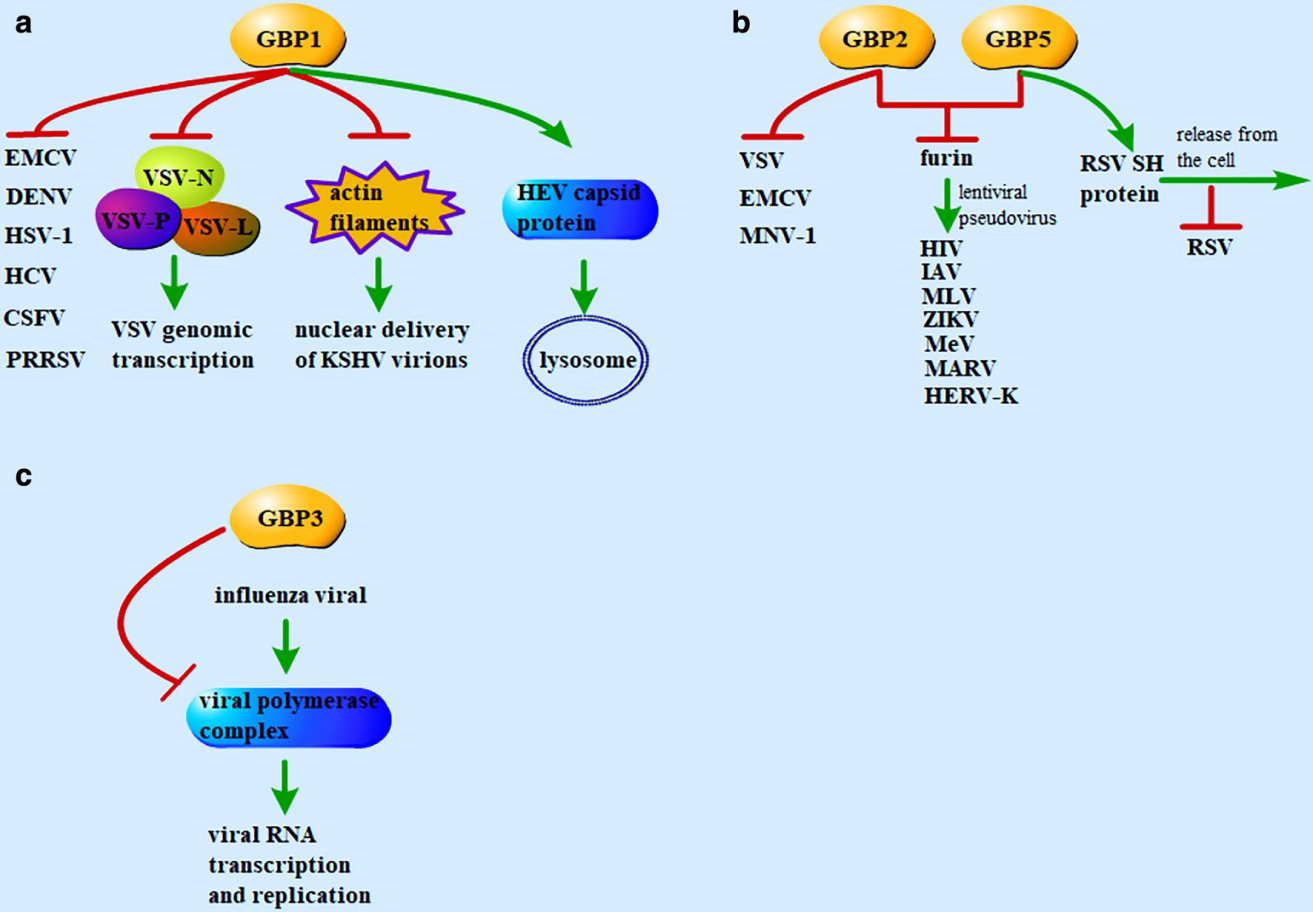
The direct antiviral functions of GBPs. **a** GBP1 suppresses the replication of EMCV, DENV, HSV-1, HCV, CSFV, PRRSV. GBP1 represses the genomic transcription of VSV by competitively binding to the VSV-N substituting for the VSV-P, inhibits the nuclear delivery of KSHV virions by disrupting the actin filaments, and inactivating the viral particle of HEV by targeting the viral capsid protein to the lysosomal compartment. **b** GBP2 inhibits the replication of VSV and EMCV, andorchestrates IFN-γ-mediated immune responses against MNV-1. GBP2 and GBP5 inhibit the replication of HIV, IAV, MLV, ZIKV, MeV, MARV and HERV-K by suppressing furin to reduce the diverse viral envelope glycoproteins. GBP5 prevents RSV replication by enhancing its SH protein release. **c** GBP3 inhibits the replication of the influenza virus by disrupting the viral polymerase complex to reduce viral RNA and protein synthesis. *EMCV* encephalomyocarditis virus, *DENV* dengue virus, *HSV-1* herpes simplex virus type 1, *HCV* hepatitis C virus, *CSFV* classical swine fever virus, *PRRSV* porcine reproduction and respiratory syndrome virus, *VSV* vesicular stomatitis virus, *VSV-N* nucleoprotein, *VSV-L* large protein, *VSV-P* phosphoprotein, *KSHV* Kaposi's sarcoma-associated herpesvirus, *HEV* hepatitis E virus, *MNV-1* murine norovirus-1, *HIV* human immunodeficiency virus-1, *IAV* influenza A virus, *MLV* murine leukemia virus, *ZIKV* Zika virus, *Mev* measles virus, *MARV* Marburg virus, *HERV-K* human endogenous retrovirus K, *RSV* respiratory syncytial virus, *SH* small hydrophobic

As a family of ISGs, several human GBPs have been reported to restrict viral infection, especially against RNA viruses. GBP1 is one of the most studied GBPs presenting antiviral effects. Early studies showed that the ectopic expression of GBP1 resulted in reduced viral progeny upon the infection of vesicular stomatitis virus (VSV), encephalomyocarditis virus (EMCV), or dengue virus, while knockdown of GBP1 facilitated viral replication [28, 29]. A recent study showed that GBP1 exhibited antiviral activity against VSV and herpes simplex virus type 1 (HSV-1) infection [30]. Nucleoprotein (VSV-N), large protein (VSV-L), and phosphoprotein (VSV-P) encoded by VSV are necessary for viral genomic transcription, among which VSV-P binds to both VSV-N and VSV-L to stimulate RNA synthesis [31, 32]. But GBP1 repressed the viral genomic transcription by competitively binding to the VSV-N substituting for the VSV-P, which was independent of its GTPase and isoprenylation activity [33] (Fig. 1a), while the inhibitory effect of GBP1 on HSV-1 is still waiting for exploration. Ectopic expression of GBP1 also suppressed the genomic replication and viral particle formation and secretion of the hepatitis C virus (HCV) [34]. Conversely, HCV nonstructural (NS) protein 5B interacted with the GTPase domain of GBP1, thus blocking its GTPase activity and antiviral effect to establish persistent infection and intracellular replication of HCV [35]. GBP1 inhibited classical swine fever virus (CSFV) replication in a similar vein depending on its GTPase activity, while CSFV NS5A counteracted the antiviral activity of GBP1 by targeting its GTPase activity [36]. Also, expression profiling and polymorphism studies suggested that GBP1 was important for host resistance against porcine reproduction and respiratory syndrome virus infection [37–40] (Fig. 1a).

GBP1 displays a peculiar feature that hydrolyses GTP to a mixture of GDP and GMP by successive cleavages, with GMP being the main product. However, it is not properly known if the oligomeric form is responsible for the stimulated activity leading to enhanced GMP formation and its effect on antiviral activity. Pandita et al. found that transition-state-induced tetramerization is associated with enhanced GMP formation supported by chimeras defective in both tetramerizations with mutant and truncated proteins. Also, ectopic expression of the mutants deficient in tetramer formation does not prevent HCV replication, suggesting the tetramer's essential antiviral functions. Their data underlines the significance of GBP1 tetramer in stimulated GMP production and demonstrates its role in the antiviral activity against HCV [41].

GBP1 also exhibited antiviral activity against Kaposi's sarcoma-associated herpesvirus (KSHV). Further study revealed that GBP1 disrupted the formation of actin filaments via its GTPase activity to disturb the natural cytoskeletal structure, which was required for the nuclear delivery of KSHV particles (Fig. 1a). However, KSHV encoded RTA, an E3 ligase, to interact with GBP1 and mediate its degradation through the ubiquitin–proteasome system [42]. In addition, a recent study showed that ectopic expression of GBP1 inhibited hepatitis E virus (HEV) replication dependent on its homodimers rather than GTPase activity, in which GBP1 dimer inactivated the viral particle by targeting the viral capsid protein to the lysosomal compartment [43] (Fig. 1a). Besides, whether GBPs inhibit the other DNA viruses is still unknown.

### GBP2 and GBP5

A previous study showed that murine GBP2 inhibited the replication of both VSV and EMCV, with the GTP binding motif being required to inhibit EMCV but not VSV [44] (Fig. 1b). Later, GBP2 and GBP5 were reported to exhibit broad antiviral activity. Ectopic expression of GBP2 and GBP5 suppressed various viruses' replication, including human immunodeficiency virus-1 (HIV-1), influenza A, murine leukemia, Zika, measles, and Marburg viruses. Knockdown of GBP2 and GBP5 led to increased production of HIV-1, and complete depletion of GBP5 also facilitated the replication of influenza A, measles, and Zika viruses. The underlying mechanism was that GBP2 and GBP5 reduced the maturation and proteolytic activity of furin to inhibit the maturation and priming of diverse viral envelope glycoproteins, and the isoprenylation- and dimerization-mediated Golgi apparatus localization but not the GTPase activity of GBP5, is critical to its antiviral function. However, lentiviral pseudoviruses were used in these experiments instead of authentic viruses [45]. Notably, GBP2 and GBP5 targeted only highly pathogenic avian influenza viruses harboring a furin cleavage site in their hemagglutinin [46, 47]. A recent study also showed that GBP2 and GBP5 suppressed furin-mediated maturation of the envelope protein of human endogenous retrovirus K [48] (Fig. 1b). However, the exact mechanism of GBPs inhibiting the host protease furin requires further exploration.

GBP5 also restricted the replication of the respiratory syncytial virus (RSV) by enhancing the release of viral small hydrophobic (SH) protein into the cell culture to decrease the intracellular SH protein level. This process depended on the isoprenylation and Golgi apparatus localization of GBP5 instead of its GTPase activity (Fig. 1b). However, RSV G protein counteracted the antiviral activity of GBP5 by upregulating the E3 ligase DZIP3 to induce K48-ubiquitination mediated degradation of GBP5 through the proteasome pathway [49].

In addition to playing wide-spectrum antiviral functions directly, GBP2 and GBP5 also involve in antiviral immune signaling pathways. GBP2 orchestrated IFN-γ-mediated immune responses against murine norovirus-1 (MNV-1) replication in mouse macrophages. Further study showed that the Arg-48 and Lys-51 residues in the N-terminal LG domain, which are important for the GTPase activity, were critical for GBP2-mediated anti-MNV-1 activity. However, viruses have developed sophisticated strategies to evade host defense. MNV-1 NS7 antagonized the antiviral activity of GBP2 [50], but the underlying mechanism requires further investigation.

**Fig. 2:**
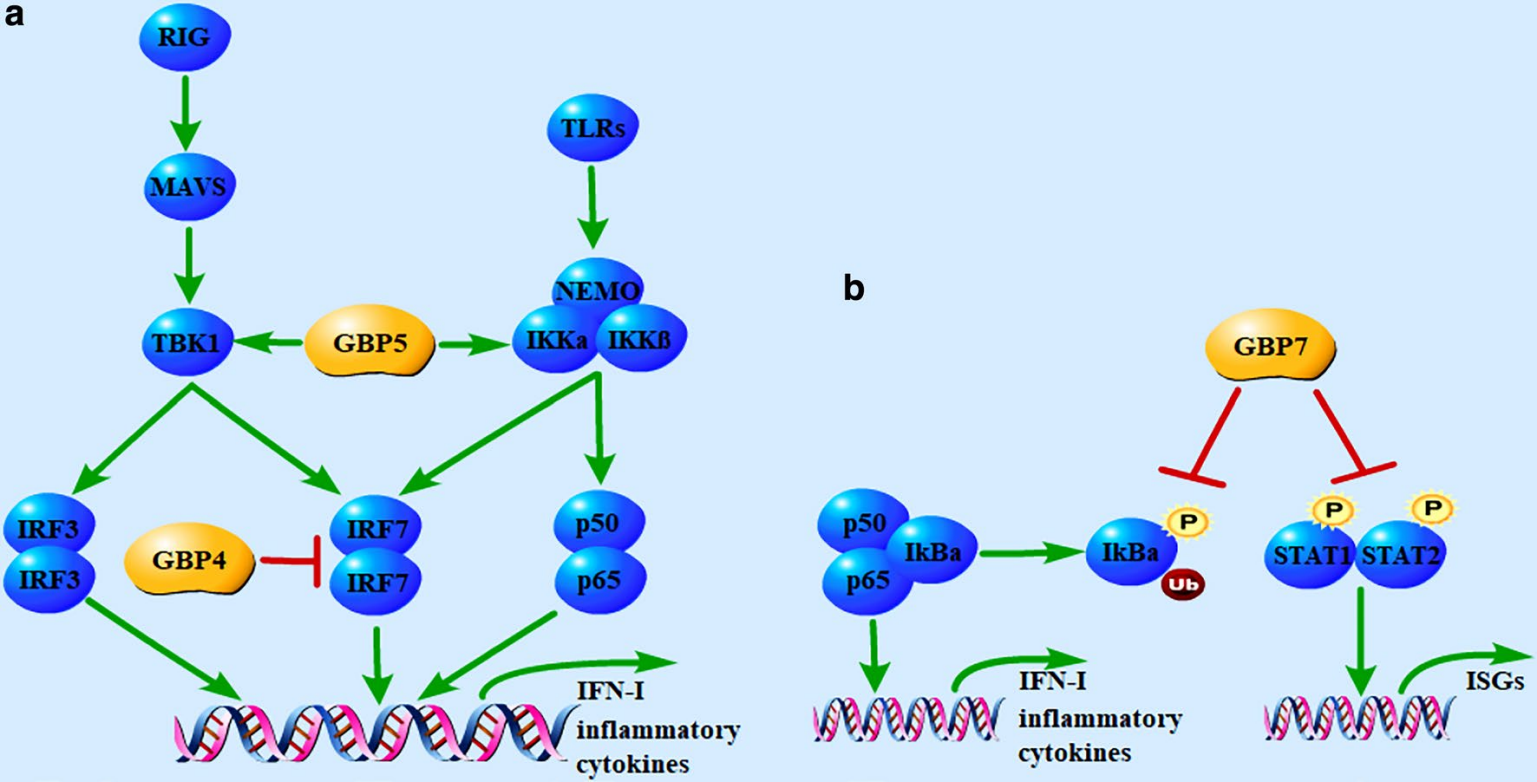
GBPs regulate host antiviral innate immune signaling pathways. **a** GBP4 negatively regulates IFN-I by impairing TRAF6-mediated ubiquitination and transactivation of IRF7. GBP5 represses replication of IAV by interacting with the NF-κB-essential modulator complex to promote IFN and proinflammatory factors expression. **b** GBP7 impedes NF-κB translocation to the nucleus by preventing the phosphorylation of IκBα and inhibits the JAK–STAT signaling pathway by attenuating the phosphorylation of STAT1 and STAT2. *P* phosphate, *Ub* ubiquitin

Feng et al. found that GBP5 was substantially upregulated in influenza patients and influenza A virus (IAV)-infected cells, and ectopic expression of GBP5 blocked viral replication. Further study revealed that GBP5 interacted with the nuclear factor-κB (NF-κB)-essential modulator complex to enhance IFN-I and proinflammatory factors production [51] (Fig. 2a).

### GBP3

GBP3 and GBP1, especially the C-terminus truncated GBP3 splice isoform (GBP3ΔC), were reported to repress the influenza virus's replication, relying on their LG domain and GTP‐binding but not hydrolysis activity. GBP3ΔC also reduced viral RNA and protein synthesis by inhibiting the viral polymerase complex [52] (Fig. 1c). Interestingly, GBP3ΔC possessed strong anti‐influenza viral activity, while the antiviral function of GBP3 was weak, indicating that the C terminus of GBP3 inhibited the antiviral activity of the LG domain.

## Proviral GBPs

### GBP4

IFN-I regulator factor 7 (IRF7) is known as an important regulator in virus-triggered IFN-I induction. Hu et al. found that virus-induced GBP4 interacted with IRF7 through its N terminal, thus disrupting the interaction between TNF receptor-associated factor 6 (TRAF6) and IRF7, which led to reduced TRAF6-mediated ubiquitination and transactivation of IRF7. Moreover, knockdown of GBP4 resulted in increased production of IFN-I triggered by virus infection. In a word, GBP4 negatively regulated virus-induced IFN-I and antiviral immunity by targeting IRF7 [53] (Fig. 2a).

### GBP7

GBP7 was demonstrated to facilitate IAV replication by inhibiting innate immune responses via NF-κB and Janus kinase-signal transducer and activator of transcription (JAK–STAT) signaling pathways. Knockout of GBP7 suppressed IAV replication by enhancing the expression of IFN-I, IFN-III, and proinflammatory cytokines, while overexpression of GBP7 facilitated viral replication. Further study revealed that GBP7 prevented the phosphorylation of IκBα to impede NF-κB translocation to the nucleus, and it also inhibited the JAK–STAT signaling pathway by attenuating the phosphorylation of STAT1 and STAT2 [54] (Fig. 2b).

## Conclusion and perspectives

The diverse roles of GBPs get involved in human diseases have been complemented by recent advances that link GBPs to inflammatory syndromes, cancers, bacterial, parasitic, and viral infections, in which the GBPs are predicated to be potent therapeutical targets [9, 55, 56]. Furthermore, GBPs are also implicated in host-virus interactions, which have now emerged as potent modulators. On the one hand, they contribute to the cellular antiviral functions by directly disrupting viral production; for example, GBP1 competes with VSV-N in binding to the VSV-P to inhibit VSV's genomic transcription, GBP1 disrupts actin filaments to inhibit the nuclear delivery of KSHV particles, GBP1 dimer targets HEV's capsid protein to the lysosomal compartment to inactivate the viral particle, GBP2 and GBP5 suppress furin to reduce the diverse viral envelope glycoproteins, GBP5 also enhances RSV SH protein release to inhibit RSV replication, GBP3 represses viral polymerase complex to reduce viral RNA and protein synthesis. On the other hand, a few GBPs control viral replication by modulating key molecules in antiviral innate immune signalings, such as GBP5 interacts with NF-κB-essential modulator complex to promote IFN and proinflammatory factors expression. However, some GBPs could promote the replication of viruses. GBP4 competes with IFR7 in binding to TRAF6 to disrupt IRF7 ubiquitination, and GBP7 not only inhibits IκBα phosphorylation to suppress nuclear translocation of NF-κB but also attenuates STAT1 and STAT2 phosphorylation to inhibit the JAK–STAT signaling pathway.

In conclusion, the GBPs family's functions in antiviral immunity may have been far underappreciated, highlighting the need for investigations to expand our understanding of GBPs biology and how to take advantage of GBPs anti- and pro-viral roles during infection and develop new antiviral therapy. We are screening the GBPs family to investigate their potential roles in antiviral innate immunity.

## Data Availability

Not applicable.

## Abbreviations

GBPs: Guanylate-binding proteins
PRRs: Pattern recognition receptors
IFN-I: Type I interferons
ISGs: Interferon-stimulated genes
GTPases: Guanosine triphosphatases
LG: Large GTPase
GTP: Guanosine-5′-triphosphate
GDP: Guanosine-5′-diphosphate
GMP: Guanosine-5′-monophosphate
LC3B: Light chain 3B
VSV: Vesicular stomatitis virus
EMCV: Encephalomyocarditis virus
HSV-1: Herpes simplex virus type 1
VSV-N: VSV’s nucleoprotein
VSV-L: VSV’s large protein
VSV-P: VSV’s phosphoprotein
HCV: Hepatitis C virus
NS: Nonstructural
CSFV: Classical swine fever virus
KSHV: Kaposi’s sarcoma-associated herpesvirus
HEV: Hepatitis E virus
HIV-1: Human immunodeficiency virus-1
RSV: Respiratory syncytial virus
SH: Small hydrophobic
MNV-1: Murine norovirus-1
IAV: Influenza A virus
NF-κB: Nuclear factor-κB
GBP3ΔC: GBP3 splice isoform
IRF7: IFN-1 regulator factor 7
TRAF6: TNF receptor-associated factor 6
JAK: Janus kinase
STAT: Signal transducer and activator of transcription

## References

[1] Wu J, Chen ZJ (2014). Innate immune sensing and signaling of cytosolic nucleic acids. Annu Rev Immunol.

[2] Ori D, Murase M, Kawai T (2017). Cytosolic nucleic acid sensors and innate immune regulation. Int Rev Immunol.

[3] Abe T, Shapira SD (2019). Negative regulation of cytosolic sensing of DNA. Int Rev Cell Mol Biol.

[4] Briard B, Place DE, Kanneganti T-D (2020). DNA sensing in the innate immune response. Physiology (Bethesda).

[5] Stanifer ML, Pervolaraki K, Boulant S (2019). Differential regulation of type I and type III interferon signaling. Int J Mol Sci.

[6] Alexopoulou L, Holt AC, Medzhitov R, Flavell RA (2001). Recognition of double-stranded RNA and activation of NF-kappaB by Toll-like receptor 3. Nature.

[7] Collins SE, Noyce RS, Mossman KL (2004). Innate cellular response to virus particle entry requires IRF3 but not virus replication. J Virol.

[8] Dempoya J, Matsumiya T, Imaizumi T, Hayakari R, Xing F, Yoshida H, Okumura K, Satoh K (2012). Double-stranded RNA induces biphasic STAT1 phosphorylation by both type I interferon (IFN)-dependent and type I IFN-independent pathways. J Virol.

[9] Pilla-Moffett D, Barber MF, Taylor GA, Coers J (2016). Interferon-inducible GTPases in host resistance, inflammation and disease. J Mol Biol.

[10] Li G, Zhang J, Sun Y, Wang H, Wang Y (2009). The evolutionarily dynamic IFN-inducible GTPase proteins play conserved immune functions in vertebrates and cephalochordates. Mol Biol Evol.

[11] Tretina K, Park ES, Maminska A, MacMicking JD (2019). Interferon-induced guanylate-binding proteins: Guardians of host defense in health and disease. J Exp Med.

[12] Praefcke GJK (2018). Regulation of innate immune functions by guanylate-binding proteins. Int J Med Microbiol.

[13] Prakash B, Praefcke GJ, Renault L, Wittinghofer A, Herrmann C (2000). Structure of human guanylate-binding protein 1 representing a unique class of GTP-binding proteins. Nature.

[14] Ghosh A, Praefcke GJ, Renault L, Wittinghofer A, Herrmann C (2006). How guanylate-binding proteins achieve assembly-stimulated processive cleavage of GTP to GMP. Nature.

[15] Vöpel T, Kunzelmann S, Herrmann C (2009). Nucleotide dependent cysteine reactivity of hGBP1 uncovers a domain movement during GTP hydrolysis. FEBS Lett.

[16] Syguda A, Bauer M, Benscheid U, Ostler N, Naschberger E, Ince S, Stürzl M, Herrmann C (2012). Tetramerization of human guanylate-binding protein 1 is mediated by coiled-coil formation of the C-terminal α-helices. FEBS J.

[17] Vöpel T, Hengstenberg CS, Peulen T-O, Ajaj Y, Seidel CAM, Herrmann C, Klare JP (2014). Triphosphate induced dimerization of human guanylate binding protein 1 involves association of the C-terminal helices: a joint double electron-electron resonance and FRET study. Biochemistry.

[18] Ngo CC, Man SM (2017). Mechanisms and functions of guanylate-binding proteins and related interferon-inducible GTPases: roles in intracellular lysis of pathogens. Cell Microbiol.

[19] Kravets E, Degrandi D, Ma Q, Peulen T-O, Klümpers V, Felekyan S, Kühnemuth R, Weidtkamp-Peters S, Seidel CA, Pfeffer K (2016). Guanylate binding proteins directly attack *Toxoplasma**gondii* via supramolecular complexes. eLife.

[20] Kim B-H, Shenoy AR, Kumar P, Bradfield CJ, MacMicking JD (2012). IFN-inducible GTPases in host cell defense. Cell Host Microbe.

[21] Meunier E, Wallet P, Dreier RF, Costanzo S, Anton L, Rühl S, Dussurgey S, Dick MS, Kistner A, Rigard M, Degrandi D, Pfeffer K, Yamamoto M, Henry T, Broz P (2015). Guanylate-binding proteins promote activation of the AIM2 inflammasome during infection with *Francisella**novicida*. Nat Immunol.

[22] Kim B-H, Chee JD, Bradfield CJ, Park E-S, Kumar P, MacMicking JD (2016). Interferon-induced guanylate-binding proteins in inflammasome activation and host defense. Nat Immunol.

[23] Kim B-H, Shenoy AR, Kumar P, Das R, Tiwari S, MacMicking JD (2011). A family of IFN-γ-inducible 65-kD GTPases protects against bacterial infection. Science (New York, NY).

[24] Modiano N, Lu YE, Cresswell P (2005). Golgi targeting of human guanylate-binding protein-1 requires nucleotide binding, isoprenylation, and an IFN-gamma-inducible cofactor. Proc Natl Acad Sci USA.

[25] Santos JC, Broz P (2018). Sensing of invading pathogens by GBPs: at the crossroads between cell-autonomous and innate immunity. J Leukoc Biol.

[26] Tripal P, Bauer M, Naschberger E, Mörtinger T, Hohenadl C, Cornali E, Thurau M, Stürzl M (2007). Unique features of different members of the human guanylate-binding protein family. J Interferon Cytokine Res.

[27] Britzen-Laurent N, Bauer M, Berton V, Fischer N, Syguda A, Reipschläger S, Naschberger E, Herrmann C, Stürzl M (2010). Intracellular trafficking of guanylate-binding proteins is regulated by heterodimerization in a hierarchical manner. PLoS ONE.

[28] Anderson SL, Carton JM, Lou J, Xing L, Rubin BY (1999). Interferon-induced guanylate binding protein-1 (GBP-1) mediates an antiviral effect against vesicular stomatitis virus and encephalomyocarditis virus. Virology.

[29] Pan W, Zuo X, Feng T, Shi X, Dai J (2012). Guanylate-binding protein 1 participates in cellular antiviral response to dengue virus. Virol J.

[30] Gu T, Yu D, Fan Y, Wu Y, Yao Y-L, Xu L, Yao Y-G (2019). Molecular identification and antiviral function of the guanylate-binding protein (GBP) genes in the Chinese tree shrew (*Tupaia**belangeri* chinesis). Dev Comp Immunol.

[31] Heinrich BS, Maliga Z, Stein DA, Hyman AA, Whelan SPJ (2018). Phase transitions drive the formation of vesicular stomatitis virus replication compartments. mBio.

[32] Kueck T, Bloyet L-M, Cassella E, Zang T, Schmidt F, Brusic V, Tekes G, Pornillos O, Whelan SPJ, Bieniasz PD (2019). Vesicular stomatitis virus transcription is inhibited by TRIM69 in the interferon-induced antiviral state. J Virol.

[33] Gu T, Yu D, Xu L, Yao Y-L, Zheng X, Yao Y-G (2021). Tupaia guanylate-binding protein 1 interacts with vesicular stomatitis virus phosphoprotein and represses primary transcription of the viral genome. Cytokine.

[34] Itsui Y, Sakamoto N, Kurosaki M, Kanazawa N, Tanabe Y, Koyama T, Takeda Y, Nakagawa M, Kakinuma S, Sekine Y, Maekawa S, Enomoto N, Watanabe M (2006). Expressional screening of interferon-stimulated genes for antiviral activity against hepatitis C virus replication. J Viral Hepat.

[35] Itsui Y, Sakamoto N, Kakinuma S, Nakagawa M, Sekine-Osajima Y, Tasaka-Fujita M, Nishimura-Sakurai Y, Suda G, Karakama Y, Mishima K, Yamamoto M, Watanabe T, Ueyama M, Funaoka Y, Azuma S, Watanabe M (2009). Antiviral effects of the interferon-induced protein guanylate binding protein 1 and its interaction with the hepatitis C virus NS5B protein. Hepatology.

[36] Li LF, Yu J, Li Y, Wang J, Li S, Zhang L, Xia SL, Yang Q, Wang X, Yu S, Luo Y, Sun Y, Zhu Y, Munir M, Qiu HJ (2016). Guanylate-binding protein 1, an interferon-induced GTPase, exerts an antiviral activity against classical swine fever virus depending on its GTPase activity. J Virol.

[37] Gol S, Estany J, Fraile LJ, Pena RN (2015). Expression profiling of the GBP1 gene as a candidate gene for porcine reproductive and respiratory syndrome resistance. Anim Genet.

[38] Niu P, Shabir N, Khatun A, Seo B-J, Gu S, Lee S-M, Lim S-K, Kim K-S, Kim W-I (2016). Effect of polymorphisms in the GBP1, Mx1 and CD163 genes on host responses to PRRSV infection in pigs. Vet Microbiol.

[39] Pena RN, Fernández C, Blasco-Felip M, Fraile LJ, Estany J (2019). Genetic markers associated with field PRRSV-induced abortion rates. Viruses.

[40] Khatun A, Nazki S, Jeong C-G, Gu S, Mattoo SUS, Lee S-I, Yang M-S, Lim B, Kim K-S, Kim B, Lee K-T, Park C-K, Lee S-M, Kim W-I (2020). Effect of polymorphisms in porcine guanylate-binding proteins on host resistance to PRRSV infection in experimentally challenged pigs. Vet Res.

[41] Pandita E, Rajan S, Rahman S, Mullick R, Das S, Sau AK (2016). Tetrameric assembly of hGBP1 is crucial for both stimulated GMP formation and antiviral activity. Biochem J.

[42] Zou Z, Meng Z, Ma C, Liang D, Sun R, Lan K (2017). Guanylate-binding protein 1 inhibits nuclear delivery of Kaposi's sarcoma-associated herpesvirus virions by disrupting formation of actin filament. J Virol.

[43] Glitscher M, Himmelsbach K, Woytinek K, Schollmeier A, Johne R, Praefcke GJK, Hildt E (2021). Identification of the interferon-inducible GTPase GBP1 as major restriction factor for the Hepatitis E virus. J Virol.

[44] Carter CC, Gorbacheva VY, Vestal DJ (2005). Inhibition of VSV and EMCV replication by the interferon-induced GTPase, mGBP-2: differential requirement for wild-type GTP binding domain. Adv Virol.

[45] Krapp C, Hotter D, Gawanbacht A, McLaren PJ, Kluge SF, Stürzel CM, Mack K, Reith E, Engelhart S, Ciuffi A, Hornung V, Sauter D, Telenti A, Kirchhoff F (2016). Guanylate binding protein (GBP) 5 is an interferon-inducible inhibitor of HIV-1 infectivity. Cell Host Microbe.

[46] Braun E, Hotter D, Koepke L, Zech F, Groß R, Sparrer KMJ, Müller JA, Pfaller CK, Heusinger E, Wombacher R, Sutter K, Dittmer U, Winkler M, Simmons G, Jakobsen MR, Conzelmann K-K, Pöhlmann S, Münch J, Fackler OT, Kirchhoff F, Sauter D (2019). Guanylate-binding proteins 2 and 5 exert broad antiviral activity by inhibiting furin-mediated processing of viral envelope proteins. Cell Rep.

[47] Hotter D, Sauter D, Kirchhoff F (2017). Guanylate binding protein 5: impairing virion infectivity by targeting retroviral envelope glycoproteins. Small GTPases.

[48] Srinivasachar Badarinarayan S, Shcherbakova I, Langer S, Koepke L, Preising A, Hotter D, Kirchhoff F, Sparrer KMJ, Schotta G, Sauter D (2020). HIV-1 infection activates endogenous retroviral promoters regulating antiviral gene expression. Nucleic Acids Res.

[49] Li Z, Qu X, Liu X, Huan C, Wang H, Zhao Z, Yang X, Hua S, Zhang W. GBP5 is an interferon-induced inhibitor of respiratory syncytial virus. J Virol. 2020;94. 10.1128/JVI.01407-20 PMC7565618 32796072

[50] Yu P, Li Y, Li Y, Miao Z, Peppelenbosch MP, Pan Q (2020). Guanylate-binding protein 2 orchestrates innate immune responses against murine norovirus and is antagonized by the viral protein NS7. J Biol Chem.

[51] Feng J, Cao Z, Wang L, Wan Y, Peng N, Wang Q, Chen X, Zhou Y, Zhu Y (2017). Inducible GBP5 mediates the antiviral response via interferon-related pathways during influenza A virus infection. J Innate Immun.

[52] Nordmann A, Wixler L, Boergeling Y, Wixler V, Ludwig S (2012). A new splice variant of the human guanylate-binding protein 3 mediates anti-influenza activity through inhibition of viral transcription and replication. FASEB J.

[53] Hu Y, Wang J, Yang B, Zheng N, Qin M, Ji Y, Lin G, Tian L, Wu X, Wu L, Sun B (2011). Guanylate binding protein 4 negatively regulates virus-induced type I IFN and antiviral response by targeting IFN regulatory factor 7. J Immunol.

[54] Feng M, Zhang Q, Wu W, Chen L, Gu S, Ye Y, Zhong Y, Huang Q, Liu S (2021). Inducible guanylate-binding protein 7 facilitates influenza A virus replication by suppressing innate immunity via NF-κB and JAK–STAT signaling pathways. J Virol.

[55] Britzen-Laurent N, Herrmann C, Naschberger E, Croner RS, Stürzl M (2016). Pathophysiological role of guanylate-binding proteins in gastrointestinal diseases. World J Gastroenterol.

[56] Kutsch M, Coers J (2020). Human guanylate binding proteins: nanomachines orchestrating host defense. FEBS J.

